# Parents as informal caregivers of children and adolescents with spinal muscular atrophy: a systematic review of quantitative and qualitative data on the psychosocial situation, caregiver burden, and family needs

**DOI:** 10.1186/s13023-022-02407-5

**Published:** 2022-07-19

**Authors:** Maja Brandt, Lene Johannsen, Laura Inhestern, Corinna Bergelt

**Affiliations:** 1 Department of Medical Psychology, University Medical Center Hamburg-Eppendorf, Martinistraße 52, 202446 Hamburg, Germany; 2 Institute for Medical Psychology, Greifswald University Medicine, Walther-Rathenau-Straße 48, 17475 Greifswald, Germany

**Keywords:** Spinal muscular atrophy, Caregiver burden, Rare disease, Psychosocial, Family needs, Quality of life, Quantitative, Qualitative, Literature review

## Abstract

**Background:**

Spinal muscular atrophy (SMA) is a rare degenerative neuromuscular disease, mostly occurring in infants and children, leading to muscle wasting and weakness, and premature death. Due to new developments of multiple disease-modifying treatments within the last years, the interest of research in patients affected by SMA increased steadily. However, the psychosocial situation of parents as informal caregivers is still rarely addressed.

**Objectives:**

This review aims to highlight quantitative and qualitative data about the psychosocial situation, caregiver burden, and needs of parents as informal caregivers for children and adolescents with SMA.

**Methods:**

A systematic literature review was performed including quantitative and qualitative original studies focusing on different psychosocial aspects and outcomes for parents of children and adolescents < 21 years of age with SMA type I–IV (PROSPERO; registration number CRD42020219020). We searched the following databases in November 2020 with a research update in August 2021: MEDLINE, CINAHL, PsycINFO and Web of Science.

**Results:**

In total, 24 articles from 23 studies were selected for inclusion (15 quantitative studies, 7 articles from 6 qualitative studies, 2 mixed methods studies). The synthesis of included studies shows multiple sources of psychosocial burden for parents of children and adolescents affected by SMA: Most studies found reduced levels of quality of life, moderate to high levels of caregiver burden and distress, as well as physical and mental health symptoms. Further, findings indicate several unmet family needs regarding information, care coordination, treatment decisions, financial support, and adequate supportive care services.

**Conclusion:**

Parents of children and adolescents with SMA face multiple sources of psychosocial stressors, caregiver burden and various unmet family needs. To unburden families, the needs of parents as caregivers should be included in integrated care paths for SMA to improve their psychosocial situation and thus their ability to care for their children and to treat or prevent physical and mental health problems due to overburdening. Future research should focus not only on quality of life and on caregiving-related burden but should also examine the clinical relevance of reported symptoms to support the implementation of adequate support services for families affected by SMA.

**Supplementary Information:**

The online version contains supplementary material available at 10.1186/s13023-022-02407-5.

## Background

Spinal muscular atrophy (SMA) is a rare autosomal recessive neurodegenerative disease, caused by homozygous disruption of the survival motor neuron 1 (SMN1) gene by deletion, conversion, or mutation and leading to muscle wasting and proximal muscle weakness [1]. With an incidence about one in 10 000 livebirths with a carrier frequency of one in 50, spinal muscular atrophy is one of the most common rare neuromuscular diseases and the primary genetic cause of deaths in infants [1, 2]. The severity of the disease is classified into clinical subtypes, which are correlated with the age of onset and the number of the remaining copies of the SMN2-gene:

SMA type I (Werdnig-Hoffmann disease) accounts for about 50–60% of all SMA cases. It is the most severe type and appears in children under the age of 6 months. Children affected by this type have hypotonia, flaccid paralysis, and are not able to sit unaided. Because of weakened muscles needed for respiration and swallowing, children with this type usually have a high risk of paradoxical breathing and respiratory infections. In addition to respiratory problems, some children with SMA type I have heart defects, both leading to an expected survival of 2 years without treatment or respiratory support [1–4].

SMA type II (30%) occurs in children between the age of seven and 18 months. Affected children usually learn to sit, but not to stand or walk. Kyphoscoliosis usually develops, requiring surgical or orthotic interventions. Like patients with type I, respiratory insufficiency is frequent, wherefore patients often die during adolescence [1].

Type III (Kugelberg-Welander disease, 10%) occurs after 18 months of live and shows profound symptom heterogeneity. Usually, it is characterised by limited motor neuron loss, the ability to walk (with support) and a normal life expectancy, but patients experience progressive impairments like scoliosis or joint contractures [1–3, 5]. Patients with type IV disease (1%) typically have onset of symptoms in adulthood, with mild motor impairment and without respiratory or nutritional problems [1].

To reliably diagnose SMA, patients with typical clinical features need to be tested for homozygous deletion or mutation of the SMN1 gene [1]. This leads to delays in diagnosis, especially in SMA types with a later onset of symptoms [6]. In order to prevent delays of diagnosis, inclusion of SMA in nationwide newborn screenings has been discussed and pilot tested in several countries for over a decade [7]. Between 2018 and 2022, most states or provinces of the US, Canada, and Australia, as well as three European countries (Germany, Norway, and Belgium) started to screen newborns [8, 9].

Until a few years ago, standards of care for SMA included most notably palliative care, (non-)invasive ventilation, and gastrostomy for severely affected patients as well as scoliosis surgery and orthotic management for all SMA types [2, 4]. Within the past years, novel and promising disease-modifying medical treatment options (nusinersen [Spinraza®], authorised in 2017, onasemnogene abeparvovec-xioi [Zolgensma®], authorised in 2020 and risdiplam [Evrysdi®], authorised in 2021 for use in the European Union) have been successfully approved for clinical use in addition to the existing standards of care [10, 11]. Although those recently approved treatment options can reduce the mortality and severity of disease progression, curing SMA is still not possible and the long-term efficacy remains unclear, leaving ethical, medical, and financial implications for affected families unanswered [3, 10]. While pressure from regulatory authorities and the SMA community to include not only patients’ but also parents’ perspectives on the impact of new treatments on their quality of life in research increases [3, 12], the majority of studies investigating quality of life in SMA focuses on patients, using the parents’ perspective only for proxy-report [12–14]. A recent review by Messina and colleagues focusing on patient- and parent-oriented tools for quality of life, daily activities, and caregiver burden in SMA identified only nine of 36 SMA-specific studies, which included an outcome for parents (caregiver burden) [12]. Existing studies on quality of life, economic and social burden in affected families show that most of the parents of children with SMA report a high financial burden (i.e., for assistive equipment), a high amount of caregiver hours per day (≥ 10 h), reduced working hours for paid work, and an impaired quality of life [15–18].

The literature on the impact of SMA on parents as informal caregivers implicates multiple sources of burden, as well as negative psychological, social, and economic effects for affected families. To the best of our knowledge, so far, no study has systematically examined and synthesised existing literature on the psychosocial impact of SMA in children and adolescents on their parents. Therefore, the aim of our study was to review qualitative and quantitative studies on caregiver burden, the psychosocial situation, and family needs of parents as informal caregivers of children and adolescents with SMA.

## Methods

This systematic literature review was conducted and reported in accordance with the items of the PRISMA checklist for systematic reviews [19], details available as supplemental material (Additional file 1). The review protocol has been registered in PROSPERO (https://www.crd.york.ac.uk/prospero/display_record.php?ID=CRD42020219020).

### Search strategy

We searched the following electronic databases in November 2020 with a research update in August 2021: MEDLINE, CINAHL, PsycINFO and Web of Science. The primary search terms ‘spinal muscular atrophy’ and ‘SMA’ were combined with keywords addressing psychosocial- and mental health-related terms (e.g., ‘depress*’, ‘distress’, ‘quality of life’) and caregiver-related terms (e.g., ‘parent*’, ‘caregiv*’). Full search strings are available as supplemental material (Additional file 2). We also searched citations and references of included studies, and relevant reviews to identify further relevant literature.

### Eligibility criteria

To be included, studies had to meet the following criteria: published in a peer-reviewed journal, in English or German language, accessibility as full text, including children and adolescents aged 21 years or younger with a SMA diagnosis type I–IV, and at least one relevant psychosocial outcome/theme for parents or legal guardians as informal caregivers. In this context, we defined ‘psychosocial’ as ‘individual psychological and social aspects […] related to individual’s social conditions, mental and emotional health’ [20]. Further, ‘caregiver burden’ was defined as ‘an individual’s subjective perception of overload in one or more of four perspectives: physical, psychological, social, and financial through the caregiving process‘ [21], with the addition of a recent concept analysis that includes not only subjective, but also objective aspects of caregiver burden [22]. Studies were included when inclusion criteria regarding sample characteristics were met in at least 70% of one sample, or if results were reported separately for relevant study subsamples (e.g., SMA-subgroup out of more heterogenous samples or disease groups).

We excluded studies focusing on other diagnoses than SMA, reporting outcomes for SMA-patients only or samples of bereaved parents. Articles based on the same study sample were included if they reported different outcomes/themes.

### Study selection

**Fig. 1:**
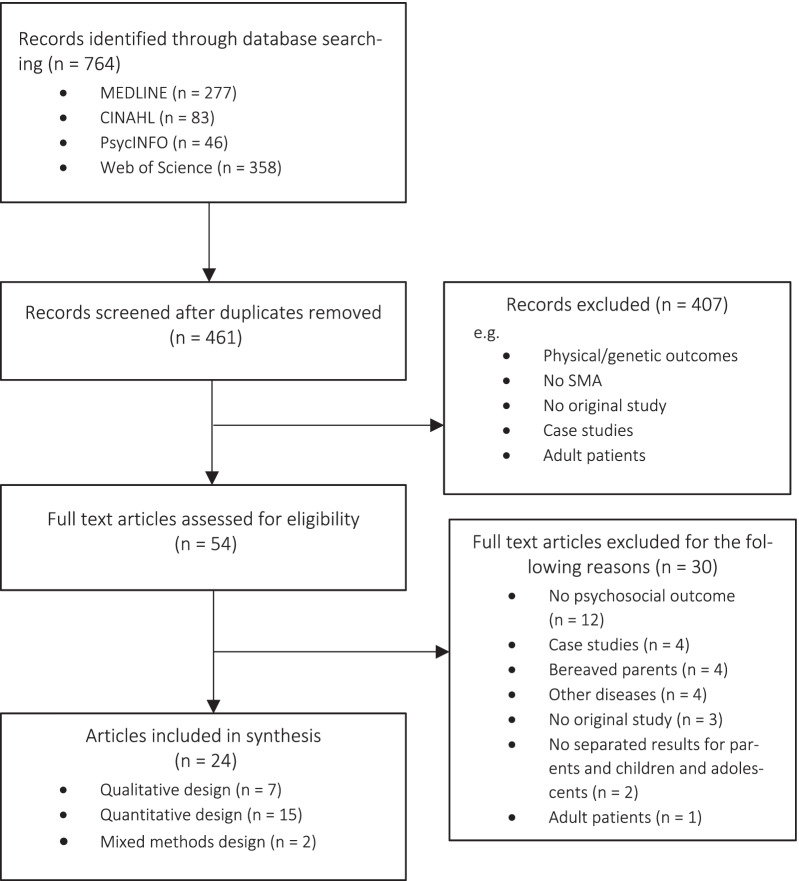
Flow diagram illustrating the selection process of included articles

The titles and abstracts of articles were screened by two independent raters (MB, LJ). Full texts were retrieved if title and abstract were of interest, or the eligibility was unclear. Studies, which seemed to meet the inclusion criteria at first, were mostly excluded because of missing psychosocial outcomes for parents (e.g., [23]), because the sample included only few cases of SMA (e.g., [24]), or because a large fraction of parents was deceased (e.g., [25]). For details of the study selection process, see Fig. 1.

### Data extraction and quality assessment

Included studies were classified by their methodological approach: (1) quantitative design, (2) qualitative design. Mixed methods studies were classified in one of the two categories, depending on the methodological approach of their relevant psychosocial outcome/aspect. Data of included studies were extracted by two of the authors (MB and LJ) independently and included the following information: citation details, country, study aims, study design, recruitment period and strategy, study population, and the main results of psychosocial outcomes (quantitative) or themes (qualitative) for parents.

To evaluate the quality of included studies, two reviewers (MB and LJ) independently assessed more than 80% (20 of 24) of the included articles using the Mixed Method Appraisal Tool (MMAT) [26]. The MMAT is a widely used instrument and has shown to be a sufficiently reliable and valid tool to appraise the methodological quality of qualitative, quantitative, and mixed methods studies [27]. The assessment of the quality of included articles revealed a good interrater agreement of 86% (Additional file 3).

### Analysis strategy

We performed a narrative analysis to synthesise the data from included articles [28]. For quantitative studies, we reported psychosocial and caregiving-related burden according to operationalisations used by the original studies. For the synthesis of qualitative findings, we decided to report ‘caregiver burden’ primarily as subjective perception of burden regarding the caregiving situation, whereas other aspects (e.g., mental, or physical health symptoms, financial costs) will be reported separately.

## Results

### Included studies

Through database search, a total of 764 records were found with 303 being duplicates. From the review of the remaining 461 articles, 54 were selected for full text screening based on their title and abstract. After reviewing full texts, 24 articles from 23 studies were selected for inclusion (15 quantitative studies, 7 articles from 6 qualitative studies, 2 mixed methods studies, see Fig. 1). The reasons for the exclusion of the remaining 30 full texts were most notably the lack of a psychosocial outcome for parents (n = 12), case studies with less than five cases of SMA (n = 4), studies with a high proportion of bereaved parents (n = 4) or other conditions than SMA (n = 4). For details see Fig. 1.

### Quantitative studies

#### Study characteristics

We identified 15 quantitative studies and one mixed methods study, including at least one psychosocial outcome for caregivers [15–17, 29–41]. For study details, see Table 1. Seven of 16 studies used parts of the same three study samples but the majority of participants in those samples were not identical and, hence, were included in our analyses as separate studies [15–17, 36–39]. Most studies were conducted in Europe and in the USA.

**Table 1:** Summary of n = 15 included studies using quantitative psychosocial outcome measures

Study	Country	Study design	Recruitment	Sample caregivers	Sample children and adolescents	Psychosocial outcomes (instruments)	Main results for psychosocial outcomes
Acar et al. [29]	Turkey	Cross-sectional, self-report paper–pencil questionnaire	July 2019– December 2019, clinic	N = 34 Sex: 68% female Age: M = 39.4 (range = 20–58) Family status: 100% married Employment: 15% of mothers were working; 97% of fathers provided a living Comparison group: none	N = 34 Sex: 74% male Age: M = 7.6 (SD = 6.2) SMA type: I = 38%, II = 38%, III = 24% Medication: NR Comparison group: none	Family needs (Family Needs Assessment Tool, FNAT), caregiver burden (Zarit Burden Interview, ZBI)	**Family needs**: Information was the most common requirement (66%), followed by financial need (46%), general support and social service (36%), and environmental disclosure (29%). **Caregiver burden**: 3% of caregivers with overload, 27% with medium/high load, 65% with light/medium load, 6% with no/little load. Moderate correlation (r = 0.57; *p* < 0.001) between caregiver burden and family needs.
Aranda-Reneo et al. [15]^a^	Spain, France, Germany, UK	Cross-sectional, self-report online-survey	July 2015–November 2015, patient organisations	N = 68 (40% Spain, 16% UK, 21% Germany, 24% France) Sex: 82% female Age: M = 39.9 (SD = 9.1) Family status: 70% married/cohabiting Employment: 60% (self-employed) Comparison groups: countries, SMA subtypes	N = 68 Sex: 53% female Age: M = 7.0 (SD = 5.2) SMA type: I = 16%, II = 62%, III = 22% Medication: NR Comparison groups: countries, SMA subtypes	Caregiver time, caregiver burden (Zarit Burden Interview, ZBI)	**Caregiver time**: M = 10.0 h (SD = 6.7) of care per day (the principal caregiver provided 6.9 h, SD = 4.6). Parents of children and adolescents with type I had a 36.3 point higher likelihood (*p* < 0.05) of providing > 10 h of daily care compared to parents of type III patients. Patients from the UK and Germany required the most time, with M = 12.5 and M = 10.6 daily hours of care. **Caregiver burden**: Total ZBI mean = 31.9 (SD = 16.5) indicating light/medium load. No significant association was found between caregiver burden and type of SMA.
Bach et al. [30]	USA	Cross-sectional study, self-report paper–pencil questionnaire	1996– September 2001, clinic	N = 84 (out of a total sample of 104 including parents of healthy controls and professional caregivers) Sex: 63% female (of parents) Age: NR Family status: NR Employment: NR Comparison group: parents of healthy children and adolescents (n = 30)	N = 46 Sex: NR Age: M = 3.0 (SD = 2.9) SMA type: I = 100% Medication: NR Comparison group: healthy children and adolescents (n = 30, mean age = 2.4)	Quality of life, effort to care for child, caregiver burden, semantic scales on how to live with affected children and adolescents (self-developed likert scales (1–10, 1–7))	**Quality of life/effort/caregiver burden**: All caregivers (incl. parents) found life with the children and adolescents to be satisfying, interesting, friendly, enjoyable, worthwhile, full, hopeful, rewarding, and estimated the children and adolescents to be happy and their lives worth living. 66% felt that their lives were rather hard than easy, and 54% reported feeling rather tied down than free. The effort of raising children and adolescents with SMA was higher compared to parents with unaffected children and adolescents. The burden of raising children and adolescents with SMA was not higher compared to healthy children and adolescents.
Chambers et al. [31]	Australia	Cross-sectional study, self-report paper–pencil questionnaire	2016–2017, clinic	N = 40 Sex: 92.5% female Age: 50% between 30 and 39 Family status: NR Employment: 50% part-time, 20% full-time, 30% unemployed Comparison groups: SMA subtypes	N = 40 Sex: 53.2% female Age: M = 9.38 (range = 1–22) SMA type: I = 10%, II = 65%, III = 25% Medication: NR Comparison group: SMA subtypes	Indirect costs for families (self-developed), caregiver quality of life (CarerQoL)	**Indirect costs for families**: Informal care was used extensively, particularly for SMA I and SMA II. Informal care was estimated with $33,000 per year, plus $39,000 per year in loss of income. Loss of income and unpaid informal care made up 24.2% and 19.8% of annual SMA healthcare costs. **Caregiver quality of life**: Most caregivers reported some/a lot of problems with at least one dimension of health, e.g., problems with own mental (84%) or physical health (78%). 78% of caregivers reported having financial problems because of care tasks. Caregiver VAS score was M = 6.1 (0 for worst well-being state and 10 for best well-being state).
Cremers et al. [32]	Netherlands	Cross-sectional study, self-report digital and paper–pencil questionnaire	December 2014– October 2015, registry and clinic	N = 38 Sex: 100% female Age: M = 39.7 (SD = 6.7) Family status: NR Employment: NR Comparison group: mothers of adult SMA patients (n = 10)	N = 38 Sex: 55.3% male Age: M = 8.9 (SD = 5.1) SMA type: I = 11%, II = 71%, III = 18% Medication: no Comparison group: adult patients with SMA (n = 10)	Caregiver burden (Caregiver Strain Index, CSI), depression and anxiety symptoms (Hospital Anxiety and Depression Scale, HADS), participation in vocational and leisure activities (Utrecht Scale of Rehabilitation-Participation, USER-P)	**Caregiver burden**: The majority of mothers of affected children and adolescents (73%) perceived high levels of caregiver burden. No significant differences were found between mothers of affected children and adolescents (M = 8.2, SD = 2.7), and adults (M = 7.8, SD = 2.7). Mothers often reported suffering from disturbed sleep and that caring was physically demanding and time-consuming. Psychological and financial consequences were reported by 1/3 of all mothers. **Depression and anxiety symptoms**: 53% of mothers with affected children and adolescents showed high depressive and anxiety symptoms (M = 13.8, SD = 8.1). Mothers of affected children and adolescents generally had higher scores, but differences were not significant. **Satisfaction**: Mothers of all patients with SMA were satisfied or very satisfied with their activities. No significant differences were found between mothers of affected children and adolescents, and adults with SMA. Multivariate analysis identified the frequency of participation in social/leisure activities as a significant predictor for caregiver burden, depression and anxiety symptoms, and satisfaction.
Ho et al. [33]	China	Cross-sectional study, self-report paper–pencil questionnaire	July 2016–March 2018, clinic	N = 12 Sex: NR Age: NR Family status: NR Employment: NR Comparison group: parents of children and adolescents with other neuromuscular disorders (n = 68)	N = 12 Sex: NR Age: M = 6.0 (range = 1–17) SMA subtypes: NR Medication: NR Comparison group: children and adolescents with other neuromuscular disorders (n = 68)	Health-related quality of life and family functioning (PedsQL™ Family Impact Module, FIM), parental stress (Parental Stress Scale, PSS)	**Health-related quality of life/family functioning**: Parents of children and adolescents with SMA reported lowest total PedsQL™FIM total scores (M = 58.2, SD = 16.8), parental quality of life (M = 49.8, SD = 23.6) and family functioning scores (M = 44.5, SD = 19.0) than other disease groups (all *p* < 0.01). PedsQL™FIM total score of the SMA cohort was worse than scores of patients with acquired brain injuries (0 for worst and 100 for best score). **Parental stress**: Parents of children and adolescents of all disease groups with a higher level of stress reported a significantly lower quality of life and poorer family functioning than those with lower levels of stress. 36.6% of all parents were in the high stress group. Moderate negative correlation with PedsQL™FIM total score (r = - 0.55).
Kariyawasam et al. [41]	Australia	Mixed method longitudinal study, 2 time points (baseline, 6 months), self-report paper–pencil	August 2018–July 2020, clinic	N = 29 Sex: 59% female Age: M = 31.0 (SD = 8.1) Family status: NR Employment: NR Comparison group: different time points (pre-post)	N = 18 Sex: 56% female Age: NR (newborns) SMA subtypes: NR Medication: nusinersen, Zolgensma® or risdiplam = 77% Comparison group: different time points (pre-post)	Caregiver quality of life (CarerQoL)	**Caregiver quality of life**: Although the screen-positive result for SMA was distressing for all parents, quality of life improved over time. CarerQoL baseline median score = 4 (SD = 1.4) versus 6-month median score = 8 (SD = 1.3, *p* < 0.001).
La Foresta et al. [34]	Italy	Longitudinal study, 3 time points (before 1st and 4th lumbar puncture (LP)), self-report paper–pencil questionnaire	December 2016 (start), clinic	N = 14 Sex: 93% female Age: NR Family status: NR Employment: NR Comparison group: different time points (pre-post)	N = 16 Sex: 69% male Age: 50% between 1 and 10 (range = 0.16–14) SMA subtypes: I = 100% Medication: nusinersen = 100% Comparison group: different time points (pre-post)	State anxiety (State-Trait Anxiety Inventory for adults, STAI)	**State anxiety**: 2 h before the 1st lumbar puncture (LP), mild anxiety levels were found in caregivers. Preoperative anxiety had increased significantly (*p* < 0.001) at the 4th LP, but significantly decreased after implementation of a self-developed psychological intervention at the 5th LP (*p* < 0.03).
López-Bastida et al. [16]^a^	Spain	Cross-sectional study, self-report online questionnaire	July 2015—November 2015, patient organisations	N = 81 Sex: 44% female, 9% male (else not reported) Age: M = 40.3 (SD = 7.3) Family Status: NR Employment: 31% employed, 20% housewife/-husband (else not reported) Comparison group: SMA subtypes	N = 81 Sex: 58% female Age: M = 7.2 (SD = 5.5) SMA subtypes: I = 10%, II = 74%, III = 16% Medication: NR Comparison group: SMA subtypes	Caregiver time (health-related quality of life (EQ-5D), caregiver burden (Zarit Burden Interview, ZBI)	**Caregiver time**: Average of 8.22 h per day. **Costs**: Average annual cost associated with SMA reached € 33,721 (SD = 38,700) in Spain. Family caregiver costs represented largest component of direct non-healthcare costs with € 21,127 (62.7% of the total cost of the disease in Spain). All costs were significantly higher for families of type II patients. **Health-related quality of life**: Mean EQ-5D index score = 0.48 (SD = 0.47; 0 health state equivalent to death and 1 state of full health). Mean EQ-5D VAS score = 69.1 (SD = 21.2; 0 worst imaginable health and 100 best imaginable health). Parents had lower quality of life means than general population (EQ-5D index score = 0.96). **Caregiver burden**: The average Zarit Burden Interview score was 35 (indicating mild to moderate burden).
McMillan et al. [35]	Canada	Cross-sectional study, self-report online questionnaire	January 2020–February 2021, patient organisations	N = 962 Sex: 57.6% female Age: M = 35.0 (IQR: 31.2–39.2) Family Status: NR Employment: 37% part-time, 28% full-time; 15% no changes in work hours, 40% reduced working hours, 19% extra unpaid leave off work/give up their job completely Comparison group: SMA subtypes	N = 962 Sex: NR Age: M = 11.7 (SD = 16.5) SMA type: I = 30%, II = 44%, III = 25% Medication: NR Comparison group: SMA subtypes	Health-related quality of life (EQ-5D), caregiver time (self-developed), caregiver burden (self-developed, Caregiver Strain Index, CSI)	**Health-related quality of life**: 65% had at least some degree of anxiety or depression, with higher percentages for parents in type I (74%) and II (69%) compared to type III (46%). Mean EQ-5D index score = 0.8 (SD = 0.2; 0 health state equivalent to death and 1 state of full health). **Caregiver time**: main caregiver and 2 other people (IQR: 1–3) provided unpaid care Median = 35 (IQR: 27–55) hours per week. **Caregiver burden**: 31–63% reported physical/psychological impact on their health due to caregiving (e.g., anxiety/depressive symptoms) CSI domains with the highest percentages: Changes in personal plans (77%), sleep being disturbed (71%), work adjustments (71%). Overall mean CSI score = 7.5 (SD = 3.3) indicating high stress and burden. Mean CSI score varied by the type of SMA: I = 6.8 (SD = 2.7), II = 8.1 (SD = 3.0), III = 7.3 (SD = 3.9).
Peña-Longobardo et al. [17]^a^	France, Germany, UK	Cross-sectional study, self-report online questionnaire	July 2015–November 2015, patient organisations	N = 56 (20% UK, 48% France, 55% Germany) Sex: 73% female Age: M = 39.9 (SD = 10.4) Family Status: NR Employment: NR Comparison group: countries	N = 86 Sex: NR Age: M = 7.03 (SD = 5.7) SMA type: I = 28%, II = 52%, III = 21% Medication: NR Comparison group: countries	Caregiver time, costs (self-developed) health-related quality of life (EQ-5D), caregiver burden (Zarit Burden Interview, ZBI)	**Caregiver time**: M = 12.5 (UK), 9.3 (FR), 10.7 (GER) hours per day **Costs**: Annual average cost associated with SMA = €54,295 (UK), €32,042 (FR), €51,983 (GER). Direct non-healthcare costs ranged between 79% and 86% of the total costs with informal care costs (by relatives) as main component. Most of the economic impact falls on families in the form of time spent on care. **Health-related quality of life**: Mean EQ-5D index score: M = 0.9 (UK), 0.4 (FR), 0.8 (GER; 0 health state equivalent to death and 1 state of full health) EQ-5D VAS score: M = 80 (UK), 62 (FR), 72 (GER; 0 worst imaginable health and 100 best imaginable health) **Caregiver burden**: Zarit Burden Interview score: M = 37 (UK), 21 (GER), 40 (FR), ranging from 0% mild to moderate burden (UK, GER) to 13% (FR) risk of burnout.
von Gontard et al. [36]^b^	Germany	Cross-sectional study, self-report paper–pencil questionnaire	1985 (start), clinic, patient organisations	N = 96 caregiver Sex: NR Age: NR Family Status: NR Employment: NR Comparison group: parents of healty controls (n = 59)	N = 96 children and adolescents Sex: 51% female Age: M = 11.2 (range = 6– 18) SMA type: I = 19%, II = 60%, III = 21% Medication: NR Comparison group: healthy controls (n = 59)	Parental stress (Questionnaire on Resources and Stress, QRS), social support (Fragebogen zur sozialen Unterstützung, F-SOZU), coping (Family Crisis Orientated Personal Evaluation Scale, F-COPES)	**Parental stress**: Significant differences (*p* < 0.001) between parents of SMA patients (M = 16.3, SD = 7.5) and healthy controls (M = 5.7, SD = 4.5). Families with type I and II patients are significantly more stressed than those with type III (*p* < 0.01–0.001). The greatest percentage of variance contributing to stress could be explained by the lack of social support, degree of disability and behavioral problems in the child. **Social support**: The degree of social support was significantly lower (*p* < 0.01) in SMA families (M = 4.1, SD = 0.7) compared to controls (M = 4.4, SD = 0.6). **Coping**: The coping abilities of SMA families did not differ from healthy controls on the total score or subscales of the F-COPES questionnaire.
von Gontard et al. [37]^b^	Germany	Cross-sectional study, self-report paper–pencil questionnaire	1985 (start), clinic, patient organisations	N = 46 Sex: NR Age: NR Family status: NR Employment: NR Comparison group: parents of children and adolescents with parents with fragile X syndrome (FXS) (n = 49), parents of healthy controls (n = 32)	N = 46 Sex: 100% male Age: M = 12.7 (range = 6.2— 18.1) SMA type: I = 20%, II = 52%, III = 28% Medication: NR Comparison group: children and adolescents with FXS (n = 49), healthy controls (n = 32)	Social support (Fragebogen zur sozialen Unterstützung, F-SOZU), parental stress (Questionnaire on Resources and Stress, QRS), coping (Family Crisis Orientated Personal Evaluation Scale, F-COPES)	**Parental stress**: Parental stress was significantly higher (*p* < 0.001) in FXS (M = 20.0, SD = 8.8) than in SMA families (M = 15.7, SD = 7.6) and in both compared to controls (M = 6.5, SD = 5.5). **Social support**: No inter-group differences were found regarding social support, indicating equal resources in the social network. **Coping**: No inter-group differences were found in the abilities of families to cope with their situation. No inter-group differences were found regarding familialcoping (F-COPES), except ‘mobilizing external help’ scores, which are significantly higher in the FXS than in the SMA families. High stress does not affect the SMA families’ coping abilities as much as the FXS families’. In all groups the degree of social support is lower in families with high stress, and higher when the burden is perceived to be lower.
Weaver et al. [38]^c^	USA	Longitudinal, 2 time points, self-report digital questionnaire	November 2016–September 2019, clinic	N = 35 Sex: NR Age: NR Family Status: NR Employment: NR Comparison groups: medical intervention (cohorts 1–4), subtypes	N = 35 Sex: NR Age: M = 8.0 (SD = 3.6) and M = 1.8 (SD = 0.5) years between surveys SMA type: I = 43%, II = 40%, III = 17% Medication: partly nusinersen (cohorts 2–4, 62%) Comparison groups: cohort 1 = non-treatment control, 2 = not started nusinersen, 3 = loading phase of nusinsersen, 4 = maintenance phase (cohorts 2–4 on maintenance dosing at time of the final survey), subtypes, different time points (pre-post)	Quality of life (PedsQL™ Family Impact Module, FIM)	**Quality of life**: No significant differences were found between initial and final surveys for family impact, when analyzed as a whole cross-sectional clinical population (pooling cohorts 2–4). In type II, the PedsQL™ FIM total score M (t1) = 59.2 (SD = 21.2) to M (t2) = 64.2 (SD = 21.8, *p* = 0.081) trended towards significance. In cohort 4 (maintenance dosing) significant improvements in PedsQL™FIM total score = M (t1) = 45.7 (SD = 13.0) to M (t2) = 52.6 (SD = 26.1; *p* = 0.03) were found. In type I, with M (t1) = 45.0 (SD = 12.7) to M (t2) = 50.8 (SD = 12.6; *p* = 0.05) and cohort 4 (maintenance dosing) with M (t1) = 44.5 (SD = 16.1) to M (t2) = 57.9 (SD = 23.2, *p* = 0.06) increases in the worry domain also trended toward significance (0 for worst and 100 for best score).
Weaver et al. [39]^c^	USA	Cross-sectional study, self-report digital questionnaire	November 2016–September 2019, clinic	N = 58 Sex: NR Age: NR Family Status: NR Employment: NR Comparison groups: subtypes, medical intervention	N = 58 Sex: 56.9% female Age: M = 6.1 (range = 0.3—0.2) SMA type: I = 45%, II = 40%, III = 16% Medication: nusinersen = 38% Comparison groups: subtypes, medical intervention	Quality of life (PedsQL™ Family Impact Module, FIM)	**Quality of life**: Significant differences were found between types I and II in the PedsQL™ FIM total score, parental health-related quality of life and family functioning (*p* < 0.03), indicating lower quality of life for parents of children with type I SMA. The PedsQL™ FIM total score and parental quality of life were higher for families of children not receiving nusinersen. Spinal surgery was associated with improved parental quality of life and family impact (*p* < 0.03; 0 for worst and 100 for best score).
Yao et al. [40]	China	Cross-sectional study, self-report online	March 2020, clinic	N = 101 Sex: NR Age: NR Family Status: NR Employment: NR Comparison groups: subtypes, medical intervention	N = 101 Sex: 51.5% female Age: M = 7.2 (range = 0.5–16.2) SMA type: I = 26%, II = 55%, III = 19% Medication: nusinersen = 9% Comparison groups: subtypes, medical intervention	Quality of life (PedsQL™ Family Impact Module, FIM)	**Quality of life**: Parents of children and adolescents with type III reported higher average scores in domains of physical, emotional, social, and cognitive functioning than those of children and adolescents with types I or II SMA (*p* < 0.05). Disease-related characteristics (e.g., limited mobility, stable course of disease, skeleton deformity, and digestive system dysfunction) and respiratory support were associated with lower average PedsQL™ FIM total scores (*p* < 0.05). Exercise training, multidisciplinary team management, and use of nusinersen were each associated with higher average PedsQL™ FIM total scores (*p* < 0.05; 0 for worst and 100 for best score).

*IQR* Interquartile range

^a,b,c^Studies with the same letter used parts of the same study samples

Most studies focused on affected families or patients and parents equally [29, 31, 37–40]. Five studies focused primarily on caregiver outcomes [15, 32, 33, 36, 41] and four studies assessed patients’ perspectives but, additionally, reported outcomes for parents [16, 17, 30, 34]. The majority of studies were cross-sectional, only three studies used a longitudinal design with two to three time points [34, 38, 41]. Sample sizes varied between n = 12 and n = 962 participants. No study was published before the year 2000, more than 80% were published within the last 5 years.

In almost all of the included studies, the parents were the informal caregivers of affected children and adolescents, only three studies (19%) also reported on grandparents [29, 30, 40]. In studies reporting the parents’ sex (n = 10) and age (n = 8), participants were mostly mothers as primary caregivers (44–100%). Parents’ mean age was 39–41 years in the majority of the studies (63%).

Mostly, children and adolescents included in the studies were diagnosed with SMA type II (38–75% of children and adolescents), two studies only included children and adolescents with SMA type I [30, 34]. Children and adolescents in studies including SMA subtypes I-III generally had a higher mean age than children and adolescents in studies including only type I patients (M = 6.1–12.7 vs. M = 3.0 years), whereas the sex ratio of children and adolescents was approximately balanced in most studies.

Three studies reported nusinersen and one study reported nusinersen, Zolgensma® or risdiplam as medical treatment for affected children and adolescents.

Most studies compared families within SMA subtypes (type I–III) and/or different status of medical interventions (e.g., receiving nusinersen, spinal surgery) [38–40]. Other studies compared affected families in different countries [15, 17], to healthy controls [30, 36, 37], or to families affected by other diseases [33, 37]. Further, three studies compared pre- and post-measurements before and after the start of medical treatment [34, 38, 41] and/or psychological intervention [34, 38, 41], one study compared parents of affected children and adolescents versus adult patients [32], and another study did not compare participants at all [29].

#### Outcome measures

Psychosocial and burden-related outcomes for parents were: (health-related) quality of life (n = 10), caregiver burden (n = 7), caregiver time, (indirect) costs for families, parental stress, anxiety or depression symptoms, social support, coping (n = 2) as well as family needs, and satisfaction (n = 1). All included measurements were conducted through self-assessment tools.

#### (Health-related) quality of life

The most reported outcome for parents was (health-related) quality of life, with 63% (10 of 16) of studies reporting on this measure. Four of ten studies [33, 38–40] used the PedsQL™ Family Impact Module [42], three [16, 17, 35] used the EuroQol EQ-5D-5L [43], two [31, 41] used the CarerQoL [44] and one [30] used self-developed questions.

Reduced quality of life was found in parents (a) with mental (46–84%) or physical (78%) health symptoms of their own [31, 35], (b) with financial problems due to care tasks (78%) [31], (c) of children and adolescents with more severe SMA subtypes (I–II) [33, 35, 38–40], and (d) shortly after a positive screening result for SMA [41]. Further, parents of children and adolescents with SMA were more likely to have lower quality of life than the general population [16] or parents of children and adolescents with other neuromuscular disorders [45], and if they lived in France instead of the UK or Germany [17].

Higher levels of quality of life were reported to be more likely in parents of children and adolescents with SMA type III [33, 35, 38–40], and if affected children and adolescents received spinal surgery [39], exercise training, or had a multidisciplinary team management for their treatment [40]. Nusinersen treatment was found to be either beneficial [46] or detrimental [39] for parental quality of life. One study, which used self-developed questions with semantic rating scales to measure parental quality of life, found all parents to report their lives satisfying and rewarding, even though 66% reported to have a rather hard life and 54% reported to feel tied down [30].

#### Caregiver burden

Of included studies which used caregiver burden as outcome (7 of 16), four [15–17, 29] used the Zarit Burden Interview (ZBI) [47], two [32, 35] used the Caregiver Strain Index (CSI) [48] and one [30] used self-developed questions.

In included studies using caregiver burden as an outcome, on average, most parents reported mild to moderate burden [15–17, 29], or high levels of stress due to their caregiving situation [32, 35]. The most frequently reported negative effects of caregiving on parents were sleep disturbances, physical or mental health symptoms of their own, changes in personal plans, work adjustments, and financial problems [32, 35]. One study with self-developed questions found, that although the effort to raise a child with SMA was higher than for parents with healthy children and adolescents, the feeling of burden was not [30]. One study found differences in caregiving-related burden between SMA subtypes, with the highest burden on parents of patients with type II [32]. Another study did not find any burden-related differences between parents of children and adolescents with different SMA subtypes [15].

#### Caregiver time

Four of 16 included studies used caregiver time of parents as an outcome [15–17, 35]. Caregiver time was mostly assessed using the recall method [49] to estimate daily care activities and needed time per activity with a maximum of 16 h per day for all care activities (e.g. [16]).

Included studies found an average caregiver time of at least five h per day in a Canadian sample [35] and up to 12.5 h per day in samples from the UK [15, 16], with patients suffering from more severe SMA subtypes (I–II) requiring more time (> 10 h per day) than type III patients (e.g. [15, 17]). Further, one study reported that, in addition to the main caregiver, averagely two other people were involved in the informal care of a patient with SMA and 47% received additional caregiving support by professional paid carers [35].

#### (Indirect) costs for families

Three included studies reported (indirect) costs for families that took care of a child with SMA [16, 17, 31]. All of them used self-delevoped approaches to estimate healthcare costs for affected families. Total healthcare costs varied across countries, with similar average costs per year for Australia ($33,000) [31], Spain (€33,721) [16], and France (€32,042) and higher costs for Germany (€51,983) and the UK (€54,295) [17]. All studies reported that informal care costs and loss of income, both borne by affected families, constituted substantial proportions of total healthcare costs (20–68%). Two of three studies reported extensive use of informal care, especially in families with more severe SMA types (I–II), and that families with children and adolescents suffering from SMA type II had to bear the highest average costs compared to type I and III [16, 31], although total healthcare costs for type I were reported to be even higher [31].

#### Parental stress

Three studies used parental stress as outcome, two of them [36, 37] using the Questionnaire on Resources and Stress (QRS-52) [50], and one [33] using the Parental Stress Scale (PSS) [51].

Two studies found significantly higher stress rates in parents of children and adolescents with SMA compared to parents of healthy controls [36, 37]. Within families affected by SMA, higher stress rates were found in families with less social support, more behaviorual problems of children and adolescents, and a higher degree of the child’s disability as well as for SMA type I and II compared to type III [36]. One study comparing parents of children and adolescents with different neuromuscular diseases, including SMA, reported that 37% of parents within all disease groups experienced high stress levels with significantly reduced quality of life and family functioning compared to parents with lower stress levels [33].

#### Coping

Two studies, using parts of the same study sample, assessed family coping with the Family Crisis Orientated Personal Evaluation Scale (F-COPES), reporting equal coping abilities for families affected by SMA and families with healthy children and adolescents [36, 37].

#### Social support

The German questionnaire on social support (*Fragebogen zur sozialen Unterstützung*, F-SOZU) was used in two studies, using parts of the same study sample, to measure levels of perceived social support [36, 37]. One study reported significantly lower levels of perceived social support in families affected by SMA [36], and one study found equal levels compared to families with healthy children and adolescents [37].

#### Anxiety and depression symptoms

Two studies assessed anxiety [32, 34], one of them additionally measuring depressive symptoms, using the State Anxiety Inventory (STAI) [52] and the Hospital Anxiety and Depression Scale (HADS) [53]. One study reported high anxiety and depressive symptoms in 53% of mothers of children and adolescents with SMA, indicating slightly higher symptoms compared to mothers of adult patients with SMA [32]. One study measuring anxiety levels in parents shortly before nusinersen administration via lumbar punction (LP) of affected children and adolescents showed mild anxiety symptoms which decreased over time after the introduction of a self-developed psychological intervention [34].

Additionally, one study using the subscale ‘anxiety/depression’ of the EQ-5D-5L reported that 65% of caregivers in a large Canadian sample showed at least some degree of anxiety or depressive symptoms with higher rates for parents of type I–II patients (69–74%) compared to those of type III patients (46%) [35].

#### Family needs

Family needs were assessed in one study from Turkey [29] using the Family Needs Assessment Tool (FNAT) [54]. Most parents reported needs for the subscales ‘information’ (66%), especially about institutions the child could benefit from (97%), ‘financial needs’ (46%), and ‘general support and social service’ (36%) like the need for someone to talk to about family problems (50%).

#### Satisfaction

One study comparing mothers of children and adolescents with adults with SMA assessed satisfaction with participation in daily life (e.g., vocational and leisure activities), using the Utrecht Scale of Rehabilitation-Participation (USER-P) [55]. Although all mothers reported to be (very) satisfied about their activities, multivariate analysis identified participation in social/leisure activities as a significant predictor for caregiver burden, depression and anxiety symptoms, and satisfaction.

### Qualitative studies

#### Study characteristics

Of the included studies, seven articles of six qualitative studies [56–63] and one mixed method study [64] focused at least on one psychosocial aspect of the caregivers’ experience with paediatric SMA (Table 2). Two articles shared the same study sample but had a different psychosocial focus and were therefore both included [59, 60]. Most studies (4 of 7) were conducted in Australia and in the USA, two studies are from Europe, and one is from Taiwan. Three articles focused on treatment decisions and parents’ views on new therapy options [57, 58, 62], three other articles concentrated mainly on the experience of caregiving and life with SMA more generally [56, 59, 63], and two articles focused on the experience of receiving the child’s diagnosis [60, 64]. Sample sizes varied from n = 7 to n = 64 participants. All articles were published after 2015 with data collected between 2010 and 2018.

**Table 2:** Summary of n = 8 included articles of n = 7 included studies using qualitative psychosocial themes or aspects

Study	Country	Study design	Recruitment	Sample caregivers	Sample children and adolescents	Study aim, original relevant categories for parents*^1^	Summary of identified psychosocial aspects for parents based on narrative synthesis*^2^
Farrar et al. [57]	Australia	Qualitative study, conceptual framework, semi-structured interviews in focus groups	October 2017– January 2018, clinic	N = 7 Sex: NR Age: NR Family status: NR Employment: NR	N = 7 Sex: NR Age: NR SMA type: I = 100% Medication: children of parents in 1 of 2 focus groups received nusinersen	**Aim**: to investigate processes and factors that influence treatment and healthcare decisions in SMA **Categories**: 1) hope, 2) yearning and searching, 3) patient-centered care and support, 4) community and a sense of connectedness and 5) weighing up potential treatment benefits and costs	**Experiences with treatment decision making for SMA**: Hope was most important in decision making for disease-modifying therapies. New treatment options raised hope for future healthcare and technological advances changes life with SMA. **Family experiences with and needs for care**: Caregivers were exhausted by trying to fill information gaps without discovering answers. The need for timely, coordinated and evidence-based physical, psychological and practical care and support was expressed. Input from a specialised multidisciplinary team facilitated the decision making. **Role of patient organizations**: Access to a community helped against isolation/for sharing information and experiences. Most parents viewed the SMA community as active/connected group. Some parents felt community pressure or judgment regarding treatment decisions/overwhelmed by the experiences of others.
Farrar et al. [56]	Australia	Qualitative study, interpretive phenomenological analysis, semi-structured interviews, via telephone	May 2016–June 2016, clinic	N = 7 Sex: 100% female Age: 57% between 30 and 39 (range = 30–49) Family status: 100% married Employment: 86% employed	N = 8 Sex: NR Age: M = 6.4 (range = 1–14) SMA type: II = 63%, III = 37% Medication: NR	**Aim**: to investigate the effects of SMA on the costs incurred by families, to identify gaps in provision of care **Categories**: 1) financial costs, 2) information, funding and support needs, 3) physical, mental and emotional health costs	**Costs for families**: Increased demands and stresses on family finances due to direct care costs. Balancing costs for care against needs and wants of the family. Income loss due to limited employment and career opportunities. **Caregiver burden**: Caring was ‘never ending’, generating time expenditure and fatigue. Prioritising the child’s needs ahead of own care and lack of accessibility lead to exclusion from leisure experiences. Narrowing of social networks, changes in family dynamics/relationships were reported. **Psychological and physical symptoms/effects**: Mental fatigue, stress and back pain due to lifting as well as emotional burden (e.g., concerns about the child's future health). **Family experiences with and needs for care**: Substantial time and effort navigating the system (e.g. access to information, funding etc.). Lack of defined integrated support pathways was reported. The government financial assistance only partially covered costs for families. Funding and community assistance needed strong parent advocacy/perseverance. Internet, social media, and support organizations were main information sources besides the child's doctor. The wish to improve information and supportive care services was expressed.
Kiefer et al. [58]	Germany	Qualitative longitudinal study, inductive content analysis, semi-structured interviews, 2 time points	December 2016– May 2017, clinic	N = 11 Sex: 73% female Age: NR Family status: NR Employment: NR	N = 8 Sex: 50% female Age: M = 15 months (SD = 7.7) SMA type: I = 100% Medication: nusinersen = 100%	**Aim**: to explore the experiences of caregivers whose child was diagnosed with SMA type 1 and who participated in the expanded access program (EAP) for nusinersen in Germany **Categories**: 1) life with SMA prior to the EAP, 2) life with SMA prior to the EAP, 3) experiences after enrollment in the EAP, 4) life with SMA with treatment with nusinersen 5) supporting and aggravating factors influencing the adjustment process, 6) solidarity and justice	**Parents’ experiences with SMA (EAP)**: Caregivers were faced with a progressive and fatal disease. Parents experienced high levels of uncertainty and concerns that the EAP would not be launched/could be too late for their child. The time between the approval of the EAP and the actual start was agonising (hope for treatment and fear of not being accepted). **Parents' experiences with SMA (after enrollment of EAP)**: When clinical situation of most patients stabilised, it had an reassuring effect on caregivers. Participation in the EAP gave caregivers hope for a positive development and more independence of child. Areas of persisting uncertainty and fear (e.g. availability/approval of nusinersen, stop if child did not show sufficient progress) were reported. Caregivers received participation in the EAP as a radical change from a limited life expectancy to new perspectives. **Supporting and aggravating factors during process**: Good (medical) information (+) versus lack of information (e.g. EAP launch, criteria for participation) (−). Good (informative) relationships with healthcare team ( +) versus lack of accountable contact persons (e.g. to get information) (−). Recurring processes in hospital treatment ( +). Concerns about treatment continuation (−).
Lawton et al. [64]	Australia	Mixed-method study, inductive content analysis, semi-structured interviews	2011, patient organisation	N = 7 (of 8 total sample including one sibling of an adult patient) Sex: 100% female Age: NR Family status: NR Employment: NR	N = 8 (of 9 total sample including one adult patient) Sex: NR Age: NR SMA type: II = 75%, III = 25% Medication: NR	**Aim**: to explore experiences of families regarding the journey of diagnosis, views on the potential for earlier diagnosis **Categories**: 1) first noticing symptoms of SMA, 2) searching for answers and receiving a diagnosis, 3) potentially misleading information from health professionals, 4) reflections on timing of the diagnosis 5) parents’ and relatives’ views on screening to diagnose SMA earlier	**Experiences of first symptoms (before diagnosis)**: Parents reported to have a gut feeling that something was wrong. They recognized symptoms with feelings of denial, stress, anxiety, worry. In the phase of seeking answers, participants were pessimistic about the future/frustrated. The prrocess to diagnosis was perceived as a protracted journey. Health professionals initially reassured them about the normal development of their child. **Experience of SMA diagnosis**: The majority were given the diagnosis by neurologists/pediatricians. Reactions to the diagnosis were mainly feelings of shock, numbness, potential rejection/denial, worry, and sadness. The majority believed that the diagnosis could have been determined earlier. **Family experiences with and needs for care**: Some parents felt the given information was potentially misleading. All participants wished to raise the awareness and provide formal education to health professionals about the symptoms of SMA. Some expressed the need for written information.
McGraw et al. [59]^a^	USA	Qualitative study, inductive and deductive grounded theory, focus groups and semi-structured interviews	June 2014– October 2014, patient organisation and clinic	N = 64 Sex: 77% female Age: NR Family status: NR Employment: NR	N = 65 Sex: NR Age: 94% between 0 and 17 years SMA type: I = 19%, II = 45%, III = 34% Medication: NR	**Aim**: to examine how patients with SMA, their caregivers, and clinicians defined meaningful change associated with treatment and to explore views about measures of motor function **Categories**: 1) defining meaningful change in motor function, 2) views on the HFMSE and ULM, 3) broad range of essential activities should not be overlooked, 4) ability to perform daily activities 5) respiratory function 6) swallowing, 7) fatigue and endurance, 8) caregiver sleep loss, 9) a global measure to assess overall change	**Caregiver burden**: Severe lack of sleep was a significant concern for parents of children with SMA types I and II. Waking up every night to help their child roll over to prevent bedsores, or to adjust the covers to prevent the child from getting too hot or cold. Parents were constantly aware of their children’s need of its body to be adjusted because they had to do it for them.
Qian et al. [66]^a^	USA	Qualitative study, inductive and deductive grounded theory, focus groups and semi-structured interviews	June 2014– October 2014, patient organisation and clinic	N = 64 Sex: 77% female Age: NR Family status: NR Employment: NR	N = 65 Sex: NR Age: 94% between 0 and 17 years SMA type: I = 19%, II = 45%, III = 34% Medication: NR	**Aim**: to examine factors that affected how families arrived at a diagnosis, parents’ views on newborn screening, and the impact of SMA on the lives of patients and their parents **Categories**: 1) arriving at a diagnosis of SMA, 2) views on newborn screening, 3) the psychosocial impact of SMA	**Experiences of first symptoms (before diagnosis)**: Ascertaining the diagnosis was a long process for many parents. Only living close to medical centers helped to receive diagnosis quickly. Paediatricians’ lack of knowledge made it difficult to ascertain diagnosis. Parent reported the tendency to defer the judgment of the physician and to set aside fears. **Experience of SMA diagnosis**: Often, physicians communicated the diagnosis in an insensitive/unhelpful manner. The style of communication of physicians was hard for families when receiving the news of diagnosis. **Caregiver burden**: Parents were confrontated with the premature death/uncertainty about future (feeling helpless and out of control). They experienced fear of loss of functional abilities/disease progress/dependence of child. They had to deal with ifficult treatment choices (e.g. invasive treatment) and with lost expectations for the child (grief and sadness) . Parents reported lack of sleep and never-ending burdens of caring for the child as well as frustration due to lack of handicapped access. Limited ability to socialise because of weakness and fatigue was reported. Parents reported financial burden due to loss of income caused by care obligations. Sometimes, moving for adequate schooling (and leaving jobs etc.) was necessary. **Family experiences with and needs for care**: Parents had to find information on their own/to advocate for additional testing and assessment. Parents reported problems with obtaining adequate support for children in public schools.
Van Kruijsbergen et al. [62]	Netherlands	Qualitative study, thematic analysis, semi-structured interviews	January–November 2018, registry	N = 19 Sex: 68% female Age: 63% between 30 and 39 Family status: NR Employment: NR	N = 13 (of total 16 with three deceased children) Sex: NR Age: range = 0–8 SMA subtypes: I = 44%, II = 31%, III = 19% Medication: 100%	**Aim**: to gain insight into parents’ perspectives about their decision making process concerning nusinersen treatment for their child **Categories**: 1) parents’ perspectives on nusinersen, 2) perspectives on a spectrum: from a biomedical to a holistic approach, 3) parents’ perceived needs, 4) parents’ perceived concerns, 5) what facilitated or hampered parents in their decision making process?	**Expectations of nusinersen treatment**: Parents hoped to offer their child a chance of managing disease/good life/to stay alive. Their focus was rather on the aim to battle the disease versus on the aim to reach good quality of life. Prolonging life expectancy, stopping the deterioration, and increasing independence was expected. The quality of life of the child was most important factor in making final decision. **Concerns about nusinersen treatment**: Parents were afraid of possible treatment complications/that side effects might occur. They worried about the child’s ability to cope with the treatment physically (treatment too intensive). They were afraid that the treatment would cause too much suffering and effort for the child. They reported that the prolongation of life increases their child's awareness/creates emotional pain. **Family experiences with and needs for care**: The expertise and communication style of the physician played a major role in decision making. The treating physician was mostly seen as main source of information. Most parents additionally searched for information on the internet. Searching for information was difficult (scattered over the internet).
Yang et al. [63]	Taiwan	Qualitative study, phenomenological method, in-depth interviews	March 2010 — March 2012, clinic	N = 19 Sex: 53% female Age: M = 43.4 (SD = 4.8) Family status: 95% married Employment: 68% employed	N = 10 Sex: 70% male Age: M = 10.9 (SD = 1.3) SMA subtypes: I, II Medication: NR	**Aim**: to probe into parents’ anticipatory loss of school-age children with Type I or II spinal muscular atrophy **Categories**: 1) enduring the helplessness and pressure of care, 2) suffering due to the child’s rare and unknown condition, 3) loss of hope and a reinforcement of the parent–child attachment, 4) avoiding the pressure of death and enriching the child’s life	**Confrontation with short life expectancy**: Parents perceived the dilemma that growing up with SMA means an early death for their child. Parents were devastated that children would never experience youth and middle age/not have a future. Families were under the pressure of impending death. Children were afraid of death/to die without parents (fear of separation). **Psychological symptoms/effects**: The child's short life expectancy caused long-term grief and loss. Parents agonised about their children’s fear of death. Many parents became depressed and anxious, taking medication to relieve stress. They often felt depressed and helpless because the child’s future was uncertain. They struggled about the inability to control the child’s symptoms. **Family conflicts**: Children were not favored by their grandparents because of SMA, causing family conflicts. Children encountered problems with self-identification and family support. Conflicts between parents and children often erupted. **Family experiences with and needs for care**: Medical treatments were based on trial and error, not on tried protocols. Doctors did not seem to follow up with the patients. Families often had more experience in taking care of their child than their physicians. The health care system did not recognise their childrens’ conditions. Physicians and parents had different opinions about child’s physical status. Parents had to handle the demise and death of their child alone.

^a^Studies using the same sample but concentrated on different psychosocial outcomes

^*^^1^Categories postulated by authors of the original study

^*^^2^Summary of relevant aspects based on narrative synthesis by the authors of this study

As in the included quantitative studies, in all but one article which also included grandparents [63], informal caregivers were parents of affected children and adolescents. The majority of interviewees was female (53–100%). Age, family status and employment were not reported in most studies (57–71%).

For children and adolescents in included studies, SMA subtypes varied across samples, with two of seven studies including SMA type I–III, whereas the rest focused on one or two of the subtypes I–III. The age of children and adolescents ranged between < 1 year and 25 years, with > 90% being younger than 18 years. The sex of children and adolescents was rarely reported (29% of studies). In three studies, children and adolescents received medical treatment (nusinersen).

#### Qualitative aspects

Qualitative findings of included articles could be divided into two main categories of psychosocial effects on caregivers: (1) family needs, caregiver burden, and psychosocial impact on parents as well as (2) experiences in the course of treatment (first symptoms, receiving the diagnosis, experiences with treatment and decision making).

#### Family needs, caregiver burden, and psychosocial impact on parents

The most prominent topic in the included articles (5 of 8 studies) was different kind of *family needs*. Parents mostly reported the *need for information* [56, 57, 62–64]. Even though many parents saw their child’s physician as the main source of information about the disease, they mostly did not feel sufficiently informed, sometimes left alone, and reported using the internet, social media groups, and support organisations as supplementary sources [56, 57, 62, 64]. Several articles reported the parents’ feeling of trying to close an information gap, which was reported to be time-consuming and frustrating because of difficult access to information and complexity [56, 57, 62]. The wish for information was not limited to information about the disease and treatment options, but also expressed in regard to supportive care services such as financial assistance, access to equipment, paid care, schooling etc. [56, 57, 62].

Further, some parents felt more experienced with the disease than the child’s physician, reporting the need for better *educated health care professionals* [62–64]. They experienced unawareness and lack of information on the healthcare professionals’ side, leading to ignorance toward first symptoms, delay of diagnosis, potentially misleading information about the disease and controversies with parents about testing and treatments [62, 64, 65].

Besides the need for information and the awareness of healthcare professionals for SMA, parents in several articles expressed their *need for coordinated and integrated care*, including not only medical treatment, but also psychological and practical care support. Parents often reported to feel left alone after the diagnosis, struggling to find the right solutions for their child, because of complex and time-consuming navigation through the health care and support system due to lack of clearly defined and integrated support pathways [56, 57, 62].

Further, families reported the need for *adequate supportive care services*, like specific schooling or services for children and adolescents with physical disabilities, but without cognitive impairment [56, 62]. Parents in one Australian study additionally addressed the need for  *financial support*, because government financial assistance only partially covered their costs for non-medical care (e.g., equipment), so that most families were additionally reliant on support from charities and fundraising [56].

*Caregiver burden* was reported by most parents in several articles (3 of 8). One central aspect was *prioritising the child’s need* ahead of own care or the needs and wants of other family members. This reached from constant awareness of the child to be moved or adjusted day and night, sacrificing leisure time to the extent of moving with the family and quitting jobs for adequate schooling for the affected child [56, 59, 60].

In addition, parents reported to be burdened by *never-ending care tasks*, causing fatigue, exhaustion, and severe lack of sleep due to limited leisure time [56, 59, 60]. *Financial burden* due to high care costs and loss of income, as well as *uncertainty about the future* because of the child’s disease progression, and *difficult treatment choices* were also addressed in two studies [56, 60].

The *psychosocial impact* of SMA on parents of affected children and adolescents included *own (mental) health problems* like back pain due to care tasks, depression, anxiety, and high levels of distress and emotional burden, *uncertainty about the future* because of the child’s disease progression, a *narrowing of social networks* due to limited leisure time or the child’s immobility, as well as *conflicts and changes in family dynamics* [56, 60, 63]. Further, *stresses on family finances* and *limited employment and career opportunities* due to caregiving were reported as negative effects by some parents [56].

#### Experiences in the course of treatment

Three articles concentrated specifically on the parents’ *experience of searching for and receiving the diagnosis* of their child [60, 63, 64]. Although many parents described an early feeling that something was wrong with their child, most of them received the diagnosis with a *delay* due to trusting false judgements of physicians, consulting many specialists before receiving the correct diagnosis or long distances to and waiting times for appointments and test results.

Parents mostly experienced the way to receive the diagnosis as an *(emotionally) protracted journey* accompanied by feelings of denial, stress, anxiety, and worry [60, 63, 64]. When receiving the diagnosis, parents were mostly in a *state of shock*, reporting numbness, denial, worry and increasing sadness [60, 63, 64], sometimes resulting in long term grief and depression due to *confrontation with the premature death of the child* and *lost expectations for a normal life* [63]. Further, some parents reported that the *insensitive and unhelpful style of communication* by the child’s physician made their situation even worse [66].

Three articles focused on parents’ expectations [57, 58, 62], two of them additionally on decision making [57, 58] and one of them concentrated on experiences with nusinersen as the first approved disease-modifying drug for SMA [58].

Most parents reported that *hope to change* the child’s disease progression, life expectancy and quality of life was the most central *expectation* for nusinersen treatment [57, 58, 62]. Further, parents expressed their wish for their child to *manage the disease* and live a *more independent and longer life*.

In spite of positive expectations for medical treatment options, *concerns* were also reported: besides the concern of *permanent cost takeover* for treatments, some parents worried about *treatment complications and side effects*, that the treatment might be too intensive for their child and would cause *pain and limited quality of life* as well as *long-term suffering and greater emotional pain* due to the child’s increasing awareness of the disease as it is living longer [57, 58].

In regard to *decision making*, besides hope for improvement of the child’s health situation and quality of life, the *relationship with the child’s treatment team* was the most important factor for parents to ease a final treatment decision [58]. Parents reported to feel supported in treatment decisions if they (a) got sufficient information, (b) had a good, informative relationship with physicians who stayed neutral toward the parents’ decision, and (c) were familiar with the treatment process.

Parents’ *perception of treatment effects* of nusinersen can be summarised as *a reassuring effect* because the clinical situation of most patients stabilised after treatment enrolment, giving them a *radical change of outlook* from a very limited life expectancy to new perspectives and hope for their child’s future [58].

## Discussion

The aim of this study was to systematically review the existing quantitative and qualitative literature on the psychosocial situation, caregiver burden, and family needs of parents as informal caregivers of children and adolescents with SMA. Results indicate that the parents’ perspective has been studied in several geographic settings and populations with a growing interest especially within the last 10 years.

By covering a broad variety in study focuses, sample sizes, outcome measurements and study quality, our review of included studies delivers a deeper and broader understanding of the psychosocial situation of parents as informal cargivers of children and adolescents affected by SMA.

Parents in the included studies reported that taking care of their chronically ill child can be fulfilling and satisfying despite the high caregiving effort for families [30]. However, results demonstrate that parents of children and adolescents with SMA experience multiple burdens and reduced quality of life, moderate to high levels of caregiver burden and stress, and often report unmet family needs (e.g., information needs, care integration and management, financial support, adequate supportive care services). Further, parents often prioritise their child’s needs over their own or over those of other family members and spend many hours per day and night to take care of their child. This is mostly associated with sacrificing leisure time and social contacts at the cost of their own quality of life as well as physical and mental health. In addition to the time-consuming day-to-day care tasks, parents spend much time and energy on closing information gaps and navigating through the healthcare and support system, trying to find suitable solutions for their child, e.g., regarding care, financial support, or adequate schooling.

Especially parents of children and adolescents with more severe SMA subtypes (I–II) are strongly affected by negative consequences of the caregiving situation: besides the necessity to spend more time on caregiving and to bear higher healthcare costs (e.g., for equipment), parents of children and adolescents with subtypes I–II are more likely to face difficult treatment decisions, ethical concerns, the premature death, or the palliative situation of their child [67–70]. These aggravating circumstances in patients affected by SMA type I-II might be associated with higher caregiver burden, reduced rates of quality of live and (mental) health problems in parental caregivers compared to parents of patients with SMA type III.

Even though not all parents might be affected by SMA in a way that calls for additional support, the findings of the included studies indicate that receiving the SMA diagnosis can cause post-traumatic symptoms and that a substantial proportion of parents experience physical and mental health problems in the course of SMA due to the caregiving situation. These findings are in line with reviews focusing on the psychosocial situation of parents with chronically ill children and adolescents with other rare diseases (e.g., cystic fibrosis, paediatric cancer) and indicate a strong recommendation for parents to receive early and ongoing assessment of their mental health needs with access to appropriate interventions to optimise parent, child, and family well-being [71–73].

Further, the vast majority of parents as primary caregivers in the included studies were mothers (44–100%). Even though this might reflect the reality of most families in which caregivers of children and adolescents with rare and/or life-limiting illnesses continue to follow the stereotypes whereby women are the predominant caregivers [72] and men are the predominant breadwinners [74], studies comparing mothers and fathers as caregivers found different levels of mental health symptoms, perceived burden, social support, and different supportive care needs [74, 75]. Therefore, our findings might be primarily applicable to mothers of children and adolescents with SMA, indicating that future research is needed to address gender differences in caregiver burden and supportive care needs between mothers and fathers of children and adolescents affected by SMA.

Due to the novelty of disease-modifying therapy options for SMA, most of the included studies were conducted before the authorisation of those therapies, leaving the question unanswered, how they could change the course of the disease and the psychosocial situation of families with children and adolescents affected by SMA. The few studies included in our review integrating parents’ experiences with new treatment options indicate that the child’s physician’s or treatment team’s expertise as well as the relationship between families and healthcare professionals might become even more important to support and help parents with difficult treatment decisions [57, 58]. The importance of the physician-parent relationship and the physician’s expertise also seems to play an essential role in the communication of diagnosis [66] as well as in palliative care decisions [69, 70]. Especially the communication of diagnosis might cause substantial emotional burden in parents of children and adolescents affected by SMA if performed in an insensitive or uninformed manner [66].

Although the review of included studies provides a deeper understanding of the caregiving situation in paediatric SMA, it also exhibits several limitations future research should try to address: Firstly, most of the included quantitative studies did not include a matched control group or SMA-specific measurements for psychosocial outcomes. Thus, as well as due to partly small sample sizes of some studies, it is difficult to assess the actual extent of caregiver burden.

Secondly, there exists a broad range of healthcare costs, access to newborn screening and disease-modifying treatments across countries, SMA subtypes and time points of data collection in the included studies, which impedes general conlusions about the caregiving situation in paediatric SMA. Although studies comparing healthcare costs and quality of life in caregivers across several European countries found higher quality of life in countries spending more money per child in healthcare costs (UK and Germany) [17], the healthcare situation in SMA is complex and rapidly changing, and therefore should be highlighted by considering more disease- and treatment-specific information in future studies.

Thirdly, essential descriptive data about caregivers was missing in most studies, making comparisons across studies difficult, and indicating that caregivers and their situation are not the main focus of the existing literature. The latter is also supported by the fact that only two of the 16 included studies using quantitative outcomes included measurements to assess clinically relevant psychological symptoms, hence neglecting the assessment of the potential need for psychological support for most caregivers despite findings of moderate to high general burden.

## Conclusions

Existing quantitative and qualitative literature about the psychosocial situation of parents as informal caregivers in paediatric SMA show multiple sources of burden including reduced levels of quality of life, moderate to high levels of stress and caregiver burden, as well as physical and mental health symptoms, especially for parents of children and adolescents affected by SMA type I and II. Further, studies show several unmet family needs regarding information, decision making, care integration, financial support, and supportive care services. To reduce the burden on families, healthcare policies should ensure sufficient financial support and adequate supportive care services, not only for affected children and adolescents, but also for parents, as well as care integration for affected families. Healthcare professionals should be educated to identify SMA-related symptoms, receive communication training for delivering life-threatening diagnoses and work in multiprofessional teams to address not only medical information needs, but also other psychosocial needs of families (e.g., psychosocial support). Further research should concentrate not only on quality of life and caregiving-related burden, but also examine the clinical relevance of symptoms reported by parents in order to prevent and, if need be, treat mental disorders due to overburdening.

## Supplementary Information

**Additional file 1**. Table with PRISMA 2020 checklist.

**Additional file 2**. Electronic database search strategy for MEDLINE, CINAHL, PsycINFO and Web of Science.

**Additional file 3.** Table with quality assessment of articles reporting a psychosocial outcome/aspect for parents of children with SMA using the MMAT.

## Data Availability

The data generated and analyzed in this study are included in the supplementary material files.

## Abbreviations

SMA: Spinal muscular atrophy
LP: Lumbar puncture
IQR: Interquartile range
