# Supplementary material for: Parents as informal caregivers of children and adolescents with spinal muscular atrophy: a systematic review of quantitative and qualitative data on the psychosocial situation, caregiver burden, and family needs
Source: Orphanet J Rare Dis. 2022 Jul 19;17:274. doi: 10.1186/s13023-022-02407-5 (PMC9295422; doi:10.1186/s13023-022-02407-5)
Supplement: Supplementary file 2 — Additional file 2. Electronic database search strategy for MEDLINE, CINAHL, PsycINFO and Web of Science. [file 13023_2022_2407_MOESM2_ESM.docx]

**Additional file 2.** Electronic database search strategy for MEDLINE, CINAHL, PsycINFO and Web of Science

("spinal muscular atrophy") AND ("depress*" OR "anxi*" OR "burden" OR "stress" OR "distress" OR "psychosocial*" OR "mental health" OR "quality of life" OR "well-being" OR "needs" OR "experienc*") AND ("caregiv*" OR "carer" OR "parent*" OR "mother*" OR "father*" OR "famil*")
