# Supplementary material for: Parents as informal caregivers of children and adolescents with spinal muscular atrophy: a systematic review of quantitative and qualitative data on the psychosocial situation, caregiver burden, and family needs
Source: Orphanet J Rare Dis. 2022 Jul 19;17:274. doi: 10.1186/s13023-022-02407-5 (PMC9295422; doi:10.1186/s13023-022-02407-5)
Supplement: Supplementary file 3 — Additional file 3. Table with quality assessment of articles reporting a psychosocial outcome/aspect for parents of children with SMA using the MMAT. [file 13023_2022_2407_MOESM3_ESM.docx]

**Additional file 3**. Table: Quality assessment of articles reporting a psychosocial outcome/aspect for parents of children with SMA (n = 24) using the MMAT

| References | Methodological approach | Appraisal of quality | | | | | | |
| --- | --- | --- | --- | --- | --- | --- | --- | --- |
|  | 1. Qualitative  (n = 7) | **S.1 Are there clear research questions?** | **S.2 Do the collected data allow to address the research questions?** | **1.1 Is the qualitative approach appropriate to answer the research question?** | **1.2. Are the qualitative data collection methods adequate to address the research question?** | **1.3. Are the findings adequately derived from the data?** | **1.4. Is the interpretation of results sufficiently substantiated by data?** | **1.5. Is there coherence between qualitative data sources, collection, analysis and interpretation?** |
| Farrar et al. 2020 |  | yes | yes | yes | yes | yes | yes | yes |
| Farrar et al. 2020 |  | yes | yes | yes | yes | yes | yes | yes |
| Kiefer et al. 2020 |  | yes | yes | yes | yes | yes | yes | yes |
| McGraw et al. 2017 |  | yes | yes | can't tell | yes | yes | yes | can't tell |
| Qian et al. 2015 |  | yes | yes | can't tell | yes | yes | yes | can't tell |
| Van Kruijsbergen et al. 2021 |  | yes | yes | yes | yes | yes | yes | yes |
| Yang et al. 2016 |  | yes | yes | yes | yes | yes | yes | yes |
|  | 3. Quantitative non- randomized  (n = 11) | **S.1 Are there clear research questions?** | **S.2 Do the collected data allow to address the research questions?** | **3.1 Are the participants representative of the target population?** | **3.2 Are measurements appropriate regarding both the outcome and intervention (or exposure)?** | **3.3 Are there complete outcome data?** | **3.4 Are the confounders accounted for in the design and analysis?** | **3.5 During the study period, is the intervention administered (or exposure occurred) as intended?** |
| Acar et al. 2021 |  | yes | yes | yes | yes | can't tell | yes | yes |
| Aranda-Reneo et al. 2020 |  | yes | yes | no | yes | can't tell | yes | yes |
| Bach et al. 2003 |  | yes | yes | no | no | can't tell | can't tell | yes |
| Cremers et al. 2019 |  | yes | can't tell | yes | yes | can't tell | yes | yes |
| Ho et al. 2021 |  | yes | yes | yes | yes | can't tell | yes | yes |
| La Foresta et al.  2018 |  | yes | no | can't tell | yes | can't tell | no | yes |
| Peña-Longobardo et al. 2020 |  | yes | yes | no | yes | can't tell | no | yes |
| von Gontard et al. 2012 |  | yes | yes | yes | yes | yes | yes | yes |
| von Gontard et al. 2002 |  | yes | yes | yes | yes | yes | yes | yes |
| Weaver et al. 2021 |  | yes | yes | yes | yes | can't tell | yes | yes |
| Weaver et al. 2020 |  | yes | yes | yes | yes | yes | yes | yes |
|  | 4. Quantitative descriptive  (n = 4) | **S.1 Are there clear research questions?** | **S.2 Do the collected data allow to address the research questions?** | **4.1 Is the sampling strategy relevant to address the research question?** | **4.2 Is the sample representative of the target population?** | **4.3 Are the measurements appropriate?** | **4.4 Is the risk of nonresponse bias low?** | **4.5 Is the statistical analysis appropriate to answer the research question?** |
| Chambers et al.  2020 |  | yes | yes | yes | no | yes | can't tell | yes |
| López-Bastida et al. 2017 |  | yes | yes | yes | no | yes | can't tell | yes |
| McMillan et al. 2021 |  | yes | yes | yes | no | yes | can't tell | yes |
| Yao et al. 2021 |  | yes | yes | yes | yes | yes | yes | yes |
|  | 5. Mixed methods (n = 2) | **S.1 Are there clear research questions?** | **S.2 Do the collected data allow to address the research questions?** | **5.1 Is there an adequate rationale for using a mixed methods design to address the research question?** | **5.2 Are the different components of the study effectively integrated to answer the research question?** | **5.3 Are the outputs of the integration of qualitative and quantitative components adequately interpreted?** | **5.4 Are divergences and inconsistencies between quantitative and qualitative results adequately addressed?** | **5.5 Do the different components of the study adhere to the quality criteria of each tradition of the methods involved?** |
| Kariyawasam et al. 2021 |  | yes | yes | yes | yes | yes | yes | yes |
| Lawton et al. 2015 |  | yes | yes | no | can't tell | yes | yes | no |
